# The impact of inhibitory mechanisms in the inner retina on spatial tuning of RGCs

**DOI:** 10.1038/srep21966

**Published:** 2016-02-24

**Authors:** Jin Y. Huang, Dario A. Protti

**Affiliations:** 1 Discipline of Biomedical Science, The University of Sydney, NSW 1825 Australia; 2 Discipline of Physiology, The University of Sydney, NSW 2006 Australia; 3 The Bosch Institute, The University of Sydney, NSW 2006 Australia

## Abstract

Spatial tuning properties of retinal ganglion cells (RGCs) are sharpened by lateral inhibition originating at both the outer and inner plexiform layers. Lateral inhibition in the retina contributes to local contrast enhancement and sharpens edges. In this study, we used dynamic clamp recordings to examine the contribution of inner plexiform inhibition, originating from spiking amacrine cells, to the spatial tuning of RGCs. This was achieved by injecting currents generated from physiologically recorded excitatory and inhibitory stimulus-evoked conductances, into different types of primate and mouse RGCs. We determined the effects of injections of size-dependent conductances in which presynaptic inhibition and/or direct inhibition onto RGCs were partly removed by blocking the activity of spiking amacrine cells. We found that inhibition originating from spiking amacrine cells onto bipolar cell terminals and onto RGCs, work together to sharpen the spatial tuning of RGCs. Furthermore, direct inhibition is crucial for preventing spike generation at stimulus offset. These results reveal how inhibitory mechanisms in the inner plexiform layer contribute to determining size tuning and provide specificity to stimulus polarity.

Many of the complex processing properties of neural networks arise from the precise arrangements of excitatory and inhibitory connections. In the retina, the spatio-temporal patterns of excitation and inhibition are critical for determining response strength, centre-surround organisation, adaptation, edge detection and directional selective responses of RGCs among other functional properties^1^,^2^,^3^. The spatial tuning of RGCs depends on lateral inhibition at the first and second synaptic layers, the outer plexiform layer (OPL) and the inner plexiform layer (IPL) respectively^4^,^5^,^6^.

Current knowledge about the sources of lateral inhibition derives from experimental approaches that are only able to show the contribution of the OPL and/or IPL in an indirect way for the following reasons. Firstly, bath application of pharmacological agents leads to changes in multiple targets in the retinal network, thus this method is unable to precisely isolate the sites of action and their contribution by measuring the spike output of RGCs. Secondly, mapping and quantification of excitatory and inhibitory synaptic inputs onto RGCs provide critical information about the spatial organisation of the inputs but do not report quantitatively on the impact of the inputs on spike output. Gain control is known to take place in the outer retina at the photoreceptor level^7^,^8^ and via horizontal cell feedback onto photoreceptors^9^,^10^,^11^. In the retina of non-primate mammals and other vertebrates, inhibitory mechanisms mediated by amacrine cells in the inner retina were shown to play an important role in both lateral inhibition and gain control^3^,^4^.

In the primate retina recordings from parasol ganglion cells initially suggested that lateral inhibition was mediated purely via feedback mechanisms in the OPL^12^,^13^. A more recent study in the primate retina, however, showed that inhibitory inputs modulate the strength and time course of responses in ON midget RGCs and depending on the spatial structure of the stimulus also in ON parasol RGCs^14^. In addition, we have recently shown that in the retina of the marmoset, a new world primate, RGCs receive spatially organised excitatory and inhibitory synaptic inputs in which excitation is spatially tuned, whilst inhibition displays either spatial tuning or summation^15^. Interestingly, most RGCs received bipolar and amacrine cell inputs that were modulated by lateral inhibition originating in the inner retina, confirming that inhibitory mechanisms in the IPL contribute to lateral inhibition in primate RGCs. Although these studies clearly showed that the relative amplitude and timing of excitatory and inhibitory inputs onto primate RGCs are spatially modulated, the functional consequences of this are not fully understood.

In the present study, we used conductances from an OFF narrow thorny RGC whose visual-evoked responses consisted of excitation and feedforward inhibition at stimulus onset and crossover excitation and inhibition at stimulus offset to determine the relative contributions of tetrodotoxin (TTX) -mediated presynaptic and direct inhibition onto RGCs to spatial tuning. First, we recorded responses to current injections composed of excitatory and inhibitory conductance waveforms derived from visually-evoked responses to stimuli of different sizes, and confirmed that these current injections recreated the classical centre-surround organisation of RGCs receptive fields. Second, we studied the responses to injections of excitatory and inhibitory conductance waveforms measured in the presence of a blocker of action potential signalling in the IPL. We found that inhibitory processes in the IPL sharpened the spatial tuning of RGCs. Inhibitory inputs onto the excitatory afferents as well as direct inhibitory input were shown to contribute to surround inhibition. Furthermore, inhibitory inputs were shown to be critical for preventing otherwise occurring spikes at stimulus offset. These results provide critical information about the extent of inner plexiform processing in shaping and controlling the retinal output.

## Results

### Injection of light-evoked excitatory and inhibitory conductances recreates spatial tuning properties of RGCs

**Figure 1:**
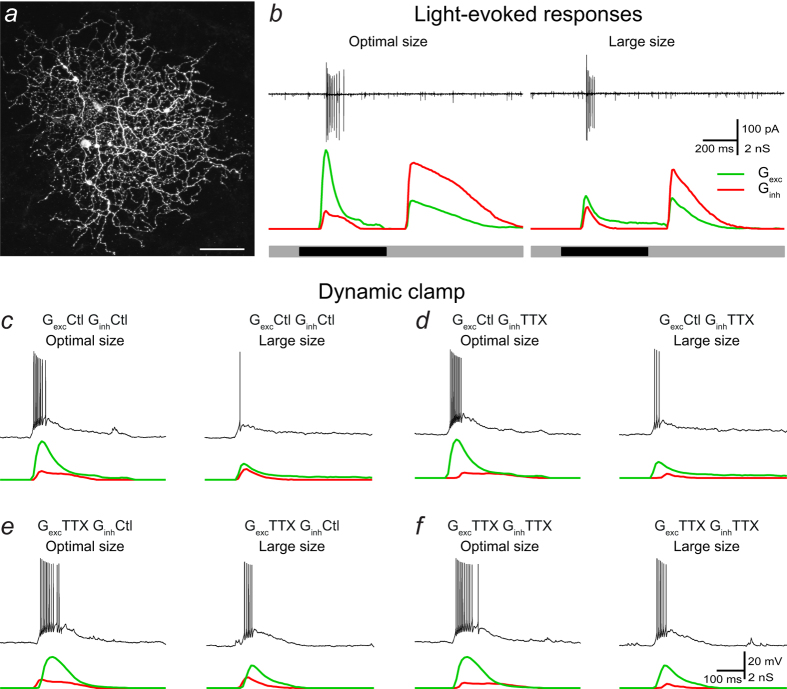
The impact of inhibitory inputs arising from spiking amacrine cells on RGC output. (**a**) Confocal stack of the narrow thorny OFF-RGC filled with Lucifer Yellow from which light-evoked responses were measured and conductances calculated. The cell body was torn away when the recording pipette was removed. Scale bar = 50 μm. (**b**) Extracellular recordings in “loose patch” mode (black traces, top) and excitatory (green traces) and inhibitory (red traces) conductances in response to decrements in light intensity from a mean luminance level with a spot diameter of 150 μm (optimal size) and with a 1400 μm diameter spot (large size). The stimulus timing is shown below the traces. Representative responses of a marmoset RGC to: (**c**) somatic injection of excitatory (green) and inhibitory (red) conductances recorded in control conditions in response to stimulation with an optimal (left) and a large (right) diameter spot; (**d**) injection of control excitatory conductance (green) and inhibitory conductance recorded in the presence of TTX (red) upon stimulation with an optimal (left) and a large (right) spot; (**e**) injection of excitatory conductance (green) recorded in the presence of TTX and inhibitory conductance recorded in control conditions (red) upon stimulation with an optimal (left) and a large (right) spot. (**f**) Injection of excitatory (green) and inhibitory (red) conductances recorded in the presence of TTX upon stimulation with an optimal (left) and a large (right) spot.

We recorded light-evoked responses from a narrow thorny RGC in the marmoset retina (Fig. 1a) in ‘loose patch’ and whole-cell voltage-clamp configurations. Extracellular recordings in ‘loose patch’ mode revealed that this cell displayed low spontaneous activity (~2 Hz) and responded to decrements in light intensity from a mean luminance level. Stimulation with a spot diameter of 150 μm (optimal size) elicited the strongest response measured as action currents and stimulation with a larger spot (1400 μm diameter, large size) produced a weaker response (Fig. 1b). Light-evoked synaptic currents were recorded in whole-cell mode after breaking in to calculate light-evoked excitatory and inhibitory synaptic conductances (Fig. 1b; see Methods). Stimulation with optimal spot size produced a large increase in excitatory conductance accompanied by an increase in inhibitory conductance, i.e. this cell displays feedforward inhibition at stimulus onset. A large increase in inhibitory conductance concomitant with a smaller increase in excitation were revealed at stimulus offset, indicating that this cell shows strong cross-over inhibition and weaker cross-over excitation as measured by its synaptic inputs. Stimulation with large spot size produced a smaller increase in excitatory conductance accompanied by an increase in inhibitory conductance at stimulus onset. Cross-over inhibition and excitation of smaller magnitude were also present at stimulus offset when stimulating with large spot diameter. To examine the role of inner plexiform inhibition mediated by spiking amacrine cells, light-evoked synaptic conductances computed from this RGC in control conditions and in the presence of the voltage-gated sodium channel blocker TTX were used in dynamic clamp recordings. In these experiments, currents generated by combining excitatory and inhibitory conductances measured in response to visual stimulation with different spot sizes in control conditions and/or in the presence of TTX were injected into RGCs of the marmoset and mouse retina. The use of these conductances allows examination of the roles of feedforward inhibition at stimulus onset and of cross-over excitation and inhibition at stimulus offset.

We recorded responses from nine marmoset RGCs, to current injection of different combinations of excitatory and inhibitory conductance waveforms measured from the above described marmoset RGC. Responses to current injections were obtained from different cell types, including midget, parasol and wide-field RGCs. Injection of control excitatory and inhibitory conductances, G_*exc*_^ctl^ and G_*inh*_^ctl^, into RGCs produced depolarising responses and action potentials. Response strength varied as a function of the spot size used to generate the conductance waveforms. Injection of conductances derived from stimulus-evoked responses generated with small spots produced relatively weak responses. Injection of conductances obtained with medium size spots produced stronger responses. Injection of conductances derived from stimulation with large spots resulted in a reduction in response strength. Figure 1c illustrates responses to current injection of conductance waveforms generated with an optimal spot and a large size spot. This response pattern shows that injection of spatially modulated excitatory and inhibitory conductance waveforms closely resembles the characteristic size-tuned responses of RGCs observed in response to stimulation with spots of different sizes.

**Figure 2:**
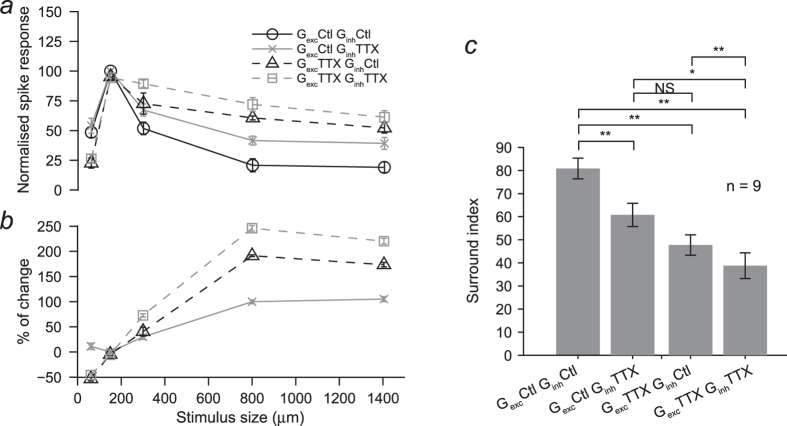
Quantification of the effect of removing TTX-mediated signal transmission in the IPL on the area-response function. Curves represent the average area-response function of nine primate RGCs. (**a**) Area-response functions of spike count normalised to their peak response in each current injection conditions tested. Note the overall reduction in surround inhibition with respect to control in all conditions evidenced by the stronger responses to injections of conductances recorded with large diameter stimuli. (**b**) The percentage of change in response strength varied with the injection of conductances recorded with stimuli of different sizes for each current injection condition. (**c**) Surround index was significantly reduced in all current injection conditions (n = 9 cells; p ≤ 0.05 Wilcoxon test). Note that the strongest effect was observed when TTX-mediated presynaptic and direct inhibition were removed.

The normalised area-response function of the spike output for injection of control conductances peaked for a spot diameter of 150 μm (optimal size), matching the peak amplitude of the excitatory conductance. Injection of excitatory and inhibitory conductances measured with larger spots resulted in the characteristic roll off of the area-response curve, indicating that they faithfully reproduce the lateral inhibition phenomenon (Fig. 2a). The suppression index (SI, see Methods) of spike output in response to injection of control conductances was 81 ± 13% (n = 9 cells). Interestingly, injection of size-tuned conductances into different cell types always rendered similar area-response functions, although some parameters such as spiking frequency and inter-spike interval varied from cell to cell, suggesting that the conductances used in this study, rather than the complement of voltage-gated channels or the structure of the cell, were the main factor in determining the shape of the area-response function.

To gain insight into the contribution of the inhibitory activity generated in the IPL to spatial tuning, we co-injected different combinations of excitatory and inhibitory conductance waveforms recorded in control conditions and during bath application of TTX, which abolishes the activity of those amacrine cells that signal via action potentials. The role of TTX-mediated direct inhibition onto RGCs was examined by injecting G_*exc*_^ctl^ and inhibitory conductances recorded in the presence of TTX (G_*inh*_^ttx^). Figure 1d shows that injection of this combination of conductance waveforms measured for optimal spot size produced a response similar to control response. Injection of conductances measured with a large spot, however, brought about stronger responses than in control conditions.

To determine the impact of the modulation of bipolar cell output by presynaptic inhibition arising from spiking amacrine cells on RGC output, we injected currents composed of excitatory conductances obtained in the presence of TTX (G_*exc*_^ttx^) and G_*inh*_^ctl^. Figure 1e shows that the response to injection of conductances measured with an optimal spot was similar to that in control conditions. Injection of conductances measured with a large spot caused a stronger response compared to control conditions.

We assessed the effects of removing inner plexiform inhibition mediated by spiking amacrine cells by injecting currents composed of G_*exc*_^ttx^ and G_*inh*_^ttx^. As shown in Fig. 1f, responses to current injection of conductances measured with optimal spot size were slightly increased with respect to injection of control conductances, and responses to injection of conductances measured with large spots were greatly increased.

The effects of removing TTX-mediated presynaptic and direct inhibition on spatial tuning are illustrated in the area-response functions for each condition normalised to the maximum spike response in each condition (Fig. 2a) and quantified by calculating their SIs. Removal of TTX-mediated direct inhibition onto RGCs resulted in responses of similar strength to control responses for optimal spot size (150 μm) and brought about an overall increase in the area-response function for injection of conductances obtained with larger size stimuli (Fig. 2a). Injection of conductances measured with spots of 300 μm diameter, which were 57% of the magnitude of the control inhibitory conductance, brought about an increase of 30% in response strength (p < 0.01). Injection of conductances measured with the largest spot diameter, for which the magnitude of the inhibitory conductance was 35% of the control inhibitory conductance, resulted in an increase of 105% of the spike output (p < 0.01). Interestingly, the first point in the area-response curves of G_*exc*_^ttx^ co-injected with G_*inh*_^ctl^ or G_*inh*_^ttx^ shows a reduction in spike response, most likely due to the decrease in G_*exc*_ elicited by the smallest spot in the presence of TTX. Responses to conductances triggered with optimal size under these conditions were similar to control values. Response strength for injection of conductances recorded with stimuli larger than the optimal size, however, was consistently increased. The area-response function normalised to the peak response in each condition shows that responses to injection of conductances of maximum size were significantly increased for all conditions compared to control (p ≤ 0.05, n = 9 cells). The change in response strength as a function of stimulus size is shown in Fig. 2b and demonstrates that the increase in response strength was larger for large stimulus sizes whilst for injection of currents generated with G_*exc*_^ttx^ of the smallest spot size there was a reduction. This clearly provides evidence that wide field spiking amacrine cells spatially modulate excitatory inputs and provide direct inhibitory input, thus, contributing to sharpen the spatial tuning of RGCs. The change in surround inhibition was quantified with SI, which was significantly reduced in all test conditions (p ≤ 0.05, n = 9 cells) as shown in Fig. 2c.

### Integration of synaptic inputs in mouse RGCs

Dynamic clamp experiments injecting currents generated with visually-evoked conductances recorded from a marmoset RGC into marmoset RGCs showed proof of the principle that the use of size tuned conductances mimics the spatial properties of RGC receptive fields. We also carried out similar current injection experiments in ganglion cells from the mouse retina to establish whether or not the response properties of mouse RGCs to different combinations of current injections showed behaviour and spatial tuning properties similar to those described above for marmoset RGCs. If established, this would provide an exciting opportunity to carry out many more experimental manipulations due to easier accessibility to mouse tissue.

**Figure 3:**
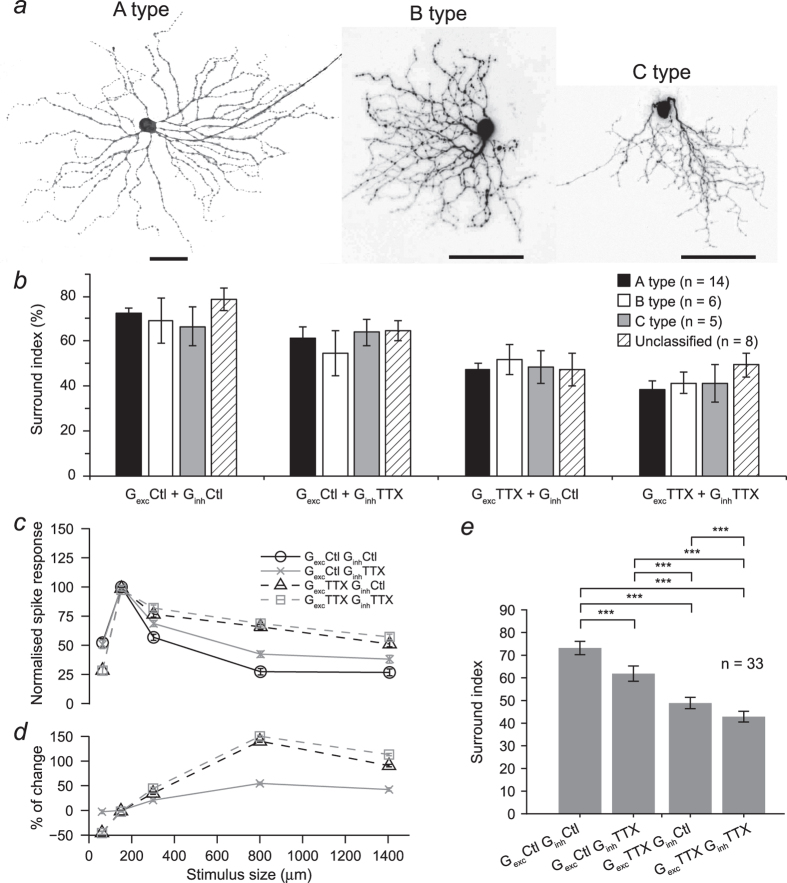
Morphology of representative mouse RGC types, quantification of the effect of removing action potential signalling in the IPL under different experimental conditions. (**a**) Morphology of representative A, B and C RGC types reconstructed from serial confocal micrographs. Scale bars: 50 μm. (**b**) Suppression indices calculated for all experimental conditions tested from the spike responses of A, B, C and unclassified RGC types. No statistically significant difference was found between any of the cell types under any of the conditions tested (Kruskal-Wallis test: range of P values was 0.44–0.94). Curves represent the average area-response function of 33 mouse RGCs. (**c**) Area-response functions of spike count normalised to their peak response in each current injection conditions tested. (**d**) The percentage of change in response strength varied with the injection of conductances recorded with stimuli of different sizes for each current injection condition. (**e**) Suppression index was significantly reduced in all current injection conditions (n = 33 cells; p ≤ 0.05 Wilcoxon test). Note that the strongest effect was observed when TTX-mediated presynaptic and direct inhibition were removed.

Given that different types of RGCs display distinct morphological, functional and intrinsic properties^16^,^17^,^18^, we first addressed the question of whether responses to current injections into different types of RGCs would result in different spatial tuning properties by analysing the responses of mouse RGCs based on cell type. Cell’s whose morphology was not fully recovered or did not clearly match Sun *et al.*’s classification^19^ were considered unclassified. We recorded from a total of 33 RGC cells in the mouse retina, of which 14 cells were A type, six B type, five C type and the remaining eight cells were unclassified. Representative examples of RGCs that were classified as group A, B or C according to soma and dendritic tree size and stratification pattern as defined in Sun *et al.* are shown in Fig. 3a. We found that response amplitude, spiking frequency and rise time varied depending on cell type (Table 1), but their responses to injection of different combinations of size tuned excitatory and inhibitory conductances showed a similar pattern in all groups, as indicated by the similarity in their SI obtained in response to each set of conductance injections (Fig. 3b).

In the following analyses we combined measurements from all RGC types, as their responses to current injections followed a similar pattern in terms of their degree of surround inhibition. The effect of abolishing the activity of spiking amacrine cells was quantified in the area-response function of responses to injection of different combinations of conductances. Figure 3c shows that injection of currents generated with excitatory conductances that were elicited with the smallest spot size in the presence of TTX resulted in a reduction in response strength. For all other conditions, TTX produced little change in the response strength at optimal size but for larger sizes it produced a significant increase in the spike output. The percentage of change in response to stimulus size shown in the bottom panel (Fig. 3d) indicates that when direct TTX-mediated inhibition is blocked there is an increase in response which then plateaus and when presynaptic inhibition is removed the response continues changing for bigger sizes. The effect of TTX-mediated inhibition on spatial tuning was quantified by calculating the SI, which shows that the degree of surround inhibition was significantly reduced in all conditions tested (p ≤ 0.001, 33 cells, Fig. 3e).

**Figure 4:**
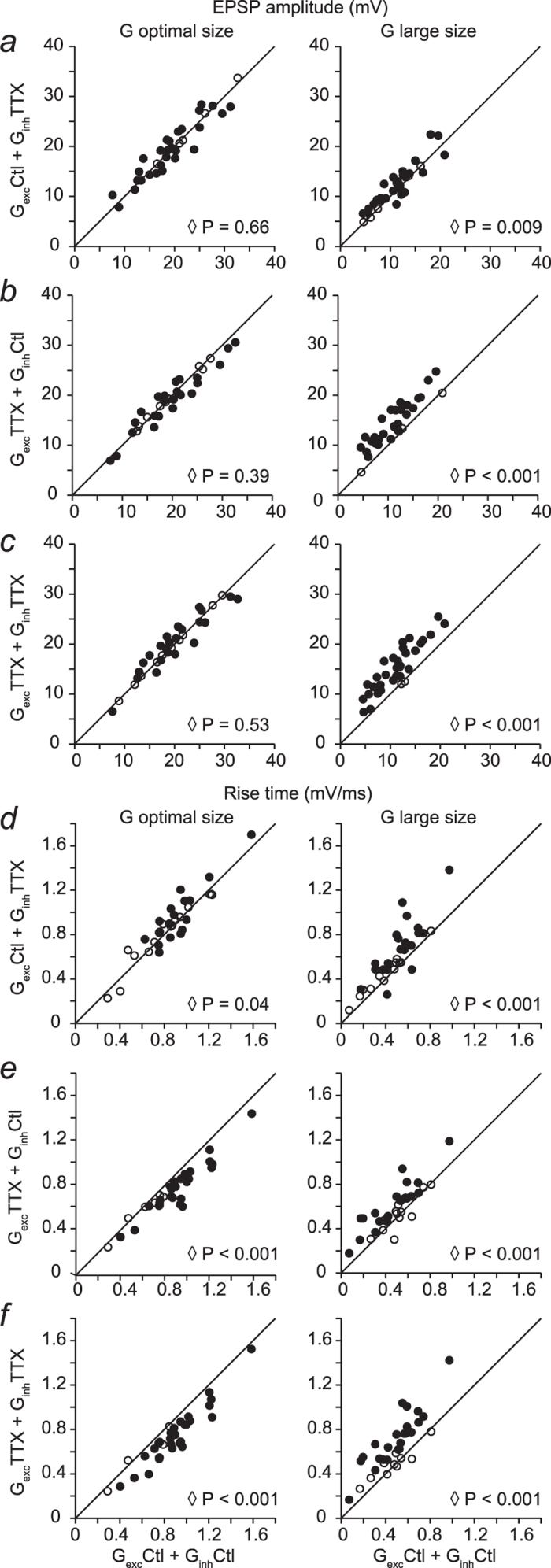
Effect of abolishing TTX-mediated signal transmission in the IPL on EPSP amplitude and rise time. Amplitude (**a–c**) and rise time (**d–f**) of excitatory post-synaptic potentials (EPSPs) for 33 mouse RGCs are plotted. X-axis: responses to injection of control excitatory and inhibitory conductances. Y-axis: responses to injection of currents generated with control excitatory and inhibitory conductance recorded in the presence of TTX (**a,d**), injection of currents generated with excitatory conductance measured in the presence of TTX and control inhibitory conductance (**b,e**) and when both excitatory and inhibitory conductances were recorded in the presence of TTX (**c,f**). Responses to conductances evoked by optimal stimulus size are shown on the left and responses to conductances evoked by large stimulus size are shown on the right. Open diamond indicates P value for the whole population, n = 33 RGCs, Wilcoxon test. Solid circles denote individual cells that exhibited significant changes between control and test conditions (p ≤ 0.03) whilst open circles correspond to individual cells that did not show significant differences (Mann–Whitney U test).

The observed changes in RGC output upon removal of TTX-mediated inner plexiform inhibition were clearly shown to depend on the relative strength of excitatory and inhibitory inputs. The underlying mechanisms that lead to increased responses in each of the conditions tested, however, are not necessarily the same: integration of these inputs might lead to an increase in the amplitude of the EPSPs, in the rate of rise of the EPSP or in both. To determine how the removal of TTX-mediated presynaptic inhibition and direct inhibitory input affect RGC output, we quantified the effects of injections of different combinations of conductances on the amplitude and rate of rise of the EPSPs. In the following we present only data obtained from mouse RGCs, although analyses of marmoset RGC recordings yielded similar results for responses to injection of conductances of large size (data not shown). Abolishing TTX-mediated direct inhibitory inputs did not modify the amplitude of EPSPs for conductances of optimal stimulus sizes and produced a small but significant (7%, p < 0.02) increase in the amplitude of EPSPs for conductances of large stimulus sizes, as seen in Fig. 4a. The rate of rise of the EPSPs was significantly increased by 24% (p < 0.001) for injection of conductances measured with large spots (Fig. 4d); this increase is likely to account for the stronger spike output observed with this combination of conductances.

Injection of excitatory conductances measured during bath application of TTX together with control inhibitory conductances produced no change in the amplitude of the EPSPs for conductances measured with optimal size spots but caused a significant increase in the EPSP amplitude for conductances measured with large size spots (28%, p < 0.001) as shown in Fig. 4b. For this combination of conductances, the rate of rise of EPSPs was reduced for conductances measured with optimal spot size and it was significantly increased for conductances measured with the largest spot size (Fig. 4e). The slowdown of EPSPs observed for optimal spots is likely to be responsible for the mild reduction in spike response with conductances of small and optimal spot sizes whilst the significant increase in amplitude and speed-up of EPSPs observed for conductances measured with large spots are responsible for the increased spike output in this condition.

Removal of TTX mediated inhibition onto bipolar cells (excitatory conductance) and directly on RGCs (inhibitory conductance) resulted in no change in the amplitude of responses evoked by injection of conductances measured with optimal spot size. For conductances recorded with spots of larger sizes, however, it led to significant increases in response amplitude (35%, p < 0.001; Fig. 4c). Interestingly, the rate of rise of the response to injection of conductances of optimal size was reduced whilst for conductances of large size it was significantly increased (34%, p < 0.001; Fig. 4f). Overall these results are consistent with the observations of enhanced responses to conductances of large size, although the reduction in rate of rise observed for G_*exc*_^ttx^ + G_*inh*_^ttx^ is not consistent with the lack of change at the spike level in this condition.

### The role of inhibitory inputs at non-preferred stimulus contrast

Crossover inhibition between the ON and OFF pathways is a common feature of the organisation of the synaptic inputs onto many types of RGCs^20^,^21^,^22^. The conductances used in our study display characteristics similar to crossover inhibition as it can be observed in the large inhibitory inputs at the offset of the response to preferred contrast that greatly exceed the magnitude of the excitatory input also present at the offset (see Methods). To determine the role of TTX-mediated inner plexiform inhibition at stimulus offset, we characterised the responses to injection of different combinations of conductances at stimulus offset.

**Figure 5:**
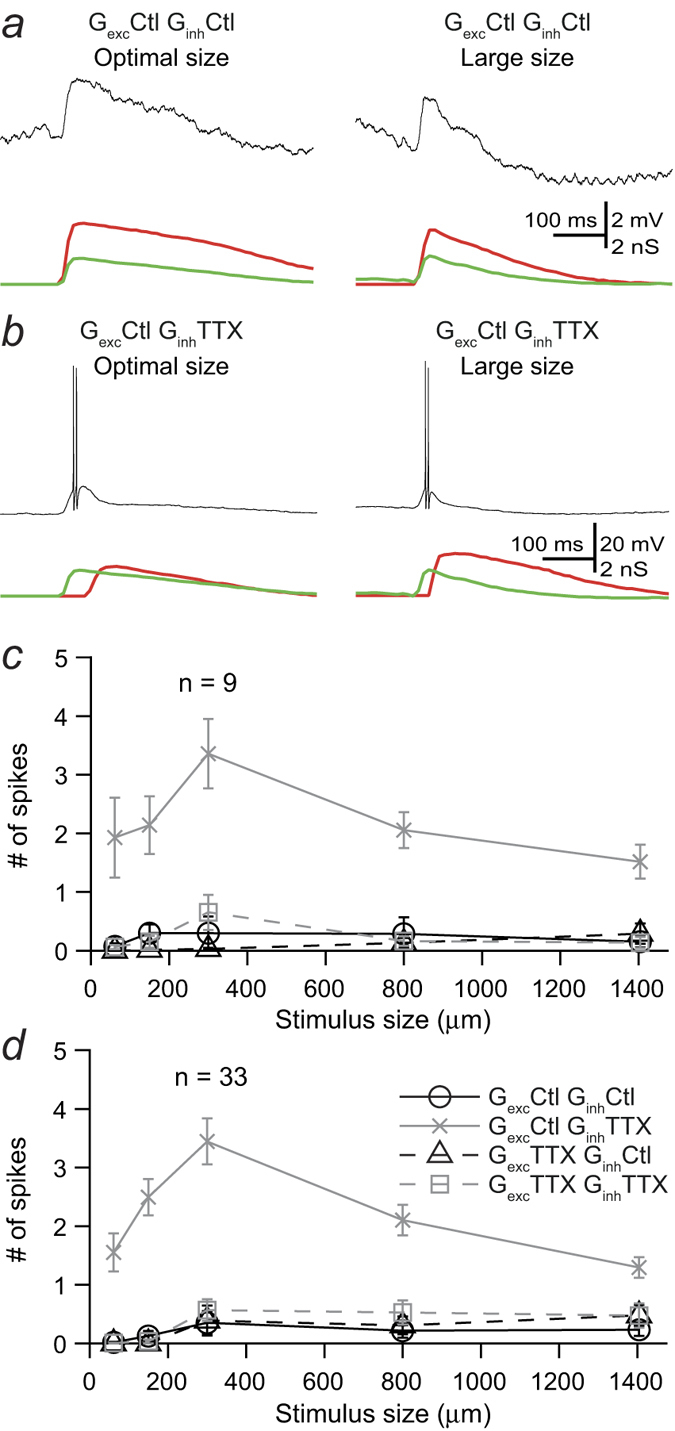
Effect of removing TTX-mediated signal transmission on the response offset. Representative responses of a marmoset RGC to: (**a**) somatic injection of excitatory (green) and inhibitory (red) conductances recorded in control conditions at the offset of stimulation with an optimal size (150 μm, left) and a large (right) diameter spot; (**b**) injection of control excitatory conductance (green) and inhibitory conductance recorded at stimulus offset in the presence of TTX (red) upon stimulation with a medium (left) and a large (right) spot; (**c**) Curves represent the average area-response function of nine primate RGCs quantified as spike count to injection of conductances recorded at stimulus offset. Note that in control conditions injection of conductances recorded at stimulus offset do not generate spike responses at any size whilst removal of TTX-mediated direct inhibitory input leads to a larger response and spikes for conductances of all sizes tested. (**d**) Average area-response function of 33 mouse RGCs in response to injection of conductances recorded at stimulus offset as in (**c**).

Injection of control conductances measured with optimal spot size (150 μm) produced small amplitude EPSPs but did not lead to spike responses (Fig. 5a). When the TTX-dependent component of the direct inhibitory input was removed, however, spike responses at stimulus offset became unmasked (Fig. 5b). Under these conditions, cells that were only responding to the onset of the stimulus in control conditions became responsive to both the onset and offset of the stimulus, behaving effectively like ON-OFF RGCs.

We quantified the effects of removing TTX-mediated inner plexiform inhibition onto bipolar cells and directly onto RGCs at non-preferred contrast stimulus on the output of RGCs. Removal of TTX-mediated inhibition on the excitatory input did not produce any significant change in spike output, as shown in the area-response functions for the injection of different combinations of conductances in marmoset (Fig. 5c) and mouse (Fig. 5d) retinae. Removal of TTX-mediated inhibitory inputs onto RGCs consistently led to spike responses for injection of currents generated with conductances of all stimulus sizes. The peak response corresponded to injection of currents generated with conductances elicited with a spot of 300 μm, which triggered the largest excitatory conductance at stimulus offset.

## Discussion

In this study we showed that inhibition in the inner plexiform layer, when present, can strongly contribute to the generation of lateral inhibition. We specifically tested the impact of inhibition originating from spiking amacrine cells, which modulate bipolar cell output and provide inhibition to ganglion cells, on the spatial tuning of RGCs. Our results demonstrate that these two loci of inhibition can independently alter the spatial tuning properties of RGCs and when acting in concert, can further enhance lateral inhibition. We also showed that crossover inhibition in RGCs with crossover excitation at the offset of preferred contrast stimulation, prevents firing in response to an otherwise non-preferred stimulus contrast.

Most previous studies have examined the sources of surround inhibition either by characterising the area-response function of the spike output and/or of the excitatory and inhibitory synaptic inputs onto RGCs before and after targeting synaptic transmission in the outer or inner plexiform layer with pharmacological agents^3^,^12^,^23^ or by modifying the activity of horizontal cells by current injection^5^. These approaches provided considerable information about the cell types and circuits mediating surround inhibition. Their interpretation, however, is not always straightforward as many drugs act on multiple cell types. For example, TTX removes inhibition on bipolar terminals but also on RGCs, GABA and glycine receptor antagonists can act on bipolar, amacrine and ganglion cells and gap-junction blockers can block hemi-channels in the OPL but also affect coupling between cells in the OPL and IPL. Our approach, which consisted of injecting currents generated with visually-evoked excitatory and inhibitory conductances via dynamic clamp recordings, allows the evaluation of the role of synaptic conductances presynaptic to RGCs separately from those impinging onto RGCs.

The effects of TTX on the injection of conductances obtained with optimal size stimuli had negligible impact on RGC output. Interestingly, injection of currents generated with excitatory conductance measured for the smallest spot (60 μm) under TTX produced a reduction in spike response. This can be explained due to the 28% reduction in peak amplitude of G_*exc*_ by TTX for this spot size. Narrow thorny RGCs stratifying in the OFF sublamina of the marmoset retina are potential targets of DB3a bipolar cells^24^, which express TTX-sensitive voltage-gated sodium channels in the macaque retina^25^. Thus, TTX is likely to reduce G_*exc*_ for small spot sizes by suppressing bipolar cell signalling whilst it increases G_*exc*_ for larger stimuli by blocking wide field, spiking amacrine cells that inhibit bipolar cells. TTX effects on the injection of conductances elicited with larger sizes, had a significant impact for all conditions tested, namely when TTX-mediated inhibition was removed from excitatory inputs and from direct inhibitory input. As expected, an even stronger impact was observed when TTX-mediated inhibition was removed from both conductances. The mechanisms by which TTX-mediated inhibition contribute to surround inhibition seem to differ when acting via inhibition of the excitatory input and via direct inhibition.

Injections of currents, generated with stimulus-evoked conductances recorded from an OFF marmoset RGC with feedforward inhibition, into different types of marmoset RGCs recreated their classical receptive field organisation with centre-surround antagonism. Interestingly, the same current injections into mouse RGCs also produced typical area-response functions with similar degrees of surround inhibition regardless of the cell type. This suggests that for cells with synaptic inputs with transient temporal properties, like those used in our study, the intrinsic properties of RGCs do not seem to be a critical factor in determining the receptive field properties. A different outcome could be expected if synaptic conductances with more sustained kinetics (i.e. ON alpha RGCs) were used in cells with a complement of membrane channels that can only account for transient responses. In this case, interpretation of the area-response function might be ambiguous due to the additional constraints of the response attributable to the intrinsic properties.

The conductances used in this study were recorded from an RGC whose synaptic inputs were spatially modulated by amacrine cells in the IPL, as shown by their sensitivity to TTX. This opens the question about how general these findings are. Several lines of evidence show that most bipolar cells show spatial tuning due to inner plexiform inhibition^3^,^15^,^26^, suggesting that inner plexiform inhibition might contribute to spatial tuning in most RGCs. Some RGCs such as the ON brisk sustained ganglion cells (BSGCs) in the rabbit retina, receive direct inhibition only via glycine receptors and this inhibitory input does not affect the centre-surround organisation of the receptive field but it modulates gain control^27^. GABAergic lateral inhibition, however, modulates excitatory inputs to BSGCs presynaptically. Therefore, even when not all RGCs respond to their preferred stimulus with an increase in direct inhibitory input (i.e. OFF alpha RGCs undergo disinhibition and an increase in excitation), presynaptic excitation seems to be spatially tuned at least partly by inner plexiform inhibition in all RGCs^3^,^15^,^27^,^28^. Our study provides unequivocal evidence of the contribution of presynaptic inhibition and direct inhibition mediated by amacrine cells to ganglion cell output.

Amacrine cell mediated inhibition from the receptive field surround is likely to modify RGC output via multiple mechanisms. Removal of TTX-mediated direct inhibition speeds up the rate of rise of postsynaptic potentials and produces a moderate yet significant increase in their amplitude. A quicker rate of rise of EPSPs is likely due to the lack of fast action potential-dependent inhibition, given that TTX produces a delay of approximately 20 ms in the onset of the inhibitory conductance. This suggests that direct inhibition most likely reduces excitability by slowing down the rising time of the postsynaptic depolarisation in addition to reducing its amplitude. A slower rising phase might also lead to stronger inactivation of sodium channels, which combined with a reduced EPSP amplitude leads to a reduction in spiking output, as we observed. When presynaptic and direct inhibition were removed, both the amplitude and rate of rise of the EPSPs were strongly enhanced. The stronger increase in EPSP amplitude indicates that presynaptic inhibition acts to reduce the output of bipolar cells whilst direct inhibition acts to slow down the EPSP.

The impact of inhibitory inputs in primate ON midget and parasol RGC spiking output has also been studied with a focus on the temporal properties of the responses using a dynamic clamp approach^14^. This study found that feed-forward inhibition elicited by full-field stimulation reduced the strength and shortened the duration of the responses in ON midget RGCs. In addition, inhibition elicited by full-field stimulation in ON parasol RGCs did not affect RGC responses. Interestingly, using a spatially structured stimulus changed the pattern of excitatory and inhibitory synaptic inputs onto ON parasol RGCs into a feed-forward arrangement in which inhibition shaped the strength and time course of the response. The conductances used in our study were obtained from an OFF primate RGC that responded to preferred stimulus with an increase in excitation concomitant with an increase in inhibition^15^. Synaptic inputs onto different RGCs are organised in different configurations: some ON and OFF primate cells can respond to preferred stimulus with an increase in excitation and feed-forward inhibition whilst other OFF RGCs respond to preferred contrast stimulus with an increase in excitation concomitant with a decrease in tonic inhibition (disinhibition)^14^,^15^,^29^,^30^. Responses in other cell types can originate via different mechanisms, for example, uniformity detectors in rabbit retina respond to changes in visual scene by decreasing their firing rate via suppression of a tonic inhibitory conductance^31^. Our finding, that direct inhibition modulates RGC response strength, is consistent with Cafaro *et al.*’s results and provides further information about the function of the size dependent magnitude of inhibition on receptive field organisation. Moreover, our study provides additional information about the functional impact of presynaptic inner retinal inhibition on the spatial organisation of RGC receptive fields.

In the visual system, lateral inhibition occurs first in the retina and it is later found in downstream areas such as the lateral geniculate nucleus^32^,^33^ and primary visual cortex^34^,^35^. Presynaptic inhibition was shown to play an important role in surround inhibition in the primary visual cortex of the cat^36^ whilst direct inhibitory input from somatostatin-expressing inhibitory neurons onto pyramidal cells contributes to surround suppression in mouse primary visual cortex^37^. Thus, both mechanisms appear to contribute to sharpening spatial tuning in different cell types and visual areas to different extents. Our results in the retina confirm that changes in the strength and/or spatial tuning of excitation and/or inhibition have a significant impact on spiking output, similar to the findings in the visual cortex.

Crossover inhibition between ON and OFF signals has been shown in different ganglion cell types using GABA blockers that target GABA receptors in bipolar cells and/or in RGCs^20^,^21^,^38^,^39^. Response polarity in some RGCs has been shown to change by a rapid shift in the image^40^ and more recently it was shown that most ON and OFF RGCs can display ON-OFF responses depending on the luminance level, suggesting that response selectivity varies as a function of light-adaptation^41^. Unmasking of crossover excitation by APB, an agonist of the metabotropic glutamate receptor mGluR6 that blocks ON bipolar cell signalling, has also been reported in ON parasol RGCs^14^,^42^. Our results confirm that inhibitory inputs at the offset of a preferred contrast stimulus are critical for providing response selectivity in cells with crossover inhibition and excitation. Elimination of TTX-mediated inhibition onto bipolar cell terminals produced only a small change as the magnitude of the excitatory conductance at stimulus offset in this condition was reduced with respect to control, most likely due to TTX-sensitivity of some bipolar cells^25^,^43^ or network effects. Removal of TTX-mediated direct inhibitory inputs onto RGGs, however, was the condition that unmasked the strongest response at stimulus offset. Interestingly we observed the strongest unmasking effect for a medium spot size, similar to previous reports showing that blockade of GABA receptors in some ON RGC types revealed OFF responses when the visual stimulus was restricted to the centre of the receptive field^39^ and in OFF alpha RGCs revealed ON responses with a similar receptive field structure as the OFF responses^21^. Our data demonstrates that some RGCs receive synaptic inputs that, when modified under different physiological or stimulation conditions, can account for switches in response polarity.

Overall, our studies provide direct evidence of the contribution of inner plexiform inhibition originating from spiking amacrine cells to the receptive field organisation and response selectivity of RGCs. In addition, they demonstrate how dynamic clamp experiments allow the dissection of the contribution of different conductances to RGC output. They also provide support for the design of experiments such as those in Cafaro *et al.* (2013) in which properties of the excitatory and inhibitory synaptic conductances can be artificially manipulated, such as their relative magnitude and/or time course, to gain further understanding of the characteristics of the synaptic inputs critical for the generation of RGC responses.

## Methods

### Marmoset tissue preparation

Recordings were made from retinae of nine adult marmosets (*Callithrix jacchus*) obtained at the end of electrophysiological experiments unrelated to this study, generously donated by Dr. Sam G. Solomon (Discipline of Physiology, The University of Sydney). Eyes were removed from the animal at the time of euthanasia under deep anaesthesia and under dim red light. The cornea, lens and vitreous were removed and the eyecup containing the sclera and retina were transferred to a dish containing carboxygenated Ames medium. A small piece of retina was isolated using a dissection microscope under white light. The piece of retina was then mounted in a recording chamber with the ganglion cells layer facing upwards. The tissue in the chamber was transferred and mounted on an upright Olympus microscope (BW50WI). The tissue was continuously perfused with carboxygenated Ames medium at a flow rate of 3–5 ml/min at 35 °C using an inline heater (Warner Instruments, TC-344B). Recordings from the ganglion cell somas, visualised through the microscope under 40x magnification using infrared illumination, were made under low ambient light conditions.

### Mouse tissue preparation

Experiments were performed on adult C57Bl/6 J mice. Animals were anesthetised by inhalation of isoflurane (Pharmachem, AU) and euthanased by cervical dislocation. Eyes were rapidly enucleated and transferred to a dish containing Ames medium where the cornea and anterior part of the eye were removed and the retinae were isolated. The remainder of the dissection procedure is similar to that described above.

All animal procedures followed the guidelines for animal experiments issued by The University of Sydney and the Australian NHMRC Code of Practice for the Care and Use of Animals for Scientific Purposes and were approved by the Animal Ethics Committee of The University of Sydney.

### Recordings

Whole-cell recordings were obtained from RGCs as previously described^44^. Borosilicate glass electrodes (6–8 MΩ) were filled with a potassium gluconate based intracellular solution containing a fluorescent dye, Lucifer Yellow. The intracellular solution contained (in mM) K^+^-gluconate: 140, MgCl_2_:4.6, EGTA: 10, HEPES: 10, ATP-Na^+^: 4 and GTP-Na^+^: 0.4. Recordings were done initially in voltage clamp mode using an EPC8 patch clamp amplifier (HEKA Elektronik, Lambrecht, Germany). Only cells that displayed large sodium currents (>2 nA) were recorded from and final confirmation of their identity was the presence of a long axon in the fluorescent and/or confocal images. Recording mode was then switched to fast current clamp mode and cells were held at -65 to -70 mV (corrected for liquid junction potential) to carry out dynamic clamp recordings.

### Dynamic clamp and properties of conductances

Conductance waveforms were obtained from voltage clamp recordings from a narrow thorny OFF-RGC in the marmoset retina (see Fig. 1a). Excitatory and inhibitory conductance waveforms were derived from responses to dark spots of different diameter on a grey background as described in detail in Di Marco *et al.*^44^. Conductances were obtained for recordings done in control conditions and in the presence of the sodium channel blocker TTX (1 μM). Each combination of excitatory and inhibitory conductances was injected 8 times. Conductances from the same marmoset RGCs were also used in the mouse experiments. Dynamic clamp experiments were carried out in carboxygenated Ames medium. Our study focused on responses to currents composed of conductances measured at stimulus onset and offset. Comparison of these responses provides critical information about the contribution of spiking amacrine cells to the excitatory and inhibitory synaptic inputs onto RGCs and to their spike output.

The spatial profile of the excitatory conductance recorded at stimulus onset in control conditions displayed surround inhibition, with a peak response for a stimulus diameter of 150 μm, which decreased as stimulus diameter was increased. The suppression index (SI, as defined below) of the excitatory conductance was 61%, indicating that surround stimulation produced a strong inhibitory effect on the centre response. The inhibitory conductance measured at stimulus onset increased with stimulus size up to 300 μm and then reached saturation. SI for the inhibitory conductance in control conditions was 8%, indicating that inhibitory inputs mostly displayed spatial summation and weak surround suppression. Bath application of TTX modified the amplitude and spatial profile of both excitatory and inhibitory stimulus-evoked conductances. The peak amplitude of excitation was reduced by 18% and the SI of the excitatory conductance during bath application of TTX was 31%. A smaller SI in this case indicates that TTX reduced inhibitory inputs onto bipolar cell terminals elicited by surround stimulation. Peak amplitude of inhibitory conductance was reduced by 46% and the SI of the inhibitory conductance was 42%. The increase in SI of the inhibitory conductance indicates that TTX removed direct inhibitory inputs onto RGCs triggered by surround stimulation.

Changes in excitatory and inhibitory conductances were also present at the offset of stimulus presentation, namely this cell displayed crossover excitation and inhibition. In control conditions excitatory and inhibitory conductances at stimulus offset were spatially tuned and showed a peak magnitude for stimuli of 300 μm and 150 μm respectively. The magnitude of the inhibitory conductance was 3.6 fold larger than that of the excitatory conductance at optimal size and on average 3.5 fold larger for all stimulus sizes. The SI of the excitatory and inhibitory conductances at stimulus offset was 59% and 57% respectively. TTX also modified the strength and spatial tuning of visually-evoked excitatory and inhibitory conductances at stimulus offset: their magnitudes were reduced by 26% and 50% respectively. The SI of the excitatory conductance was reduced to 9% and the inhibitory conductance had a SI of -48%, indicating that the magnitude of the inhibitory conductance elicited by a large spot was larger than that elicited with a spot of 300 μm.

A set up was used where a hybrid ganglion cell-computer circuit was created. Dynamic clamp was executed via a field-programmable gate arrays board (FPGA, National Instruments) using a custom-written program in Labview as described in detail in Huang *et al.*^45^. This system alters the membrane conductance of the recorded neuron based on synaptic conductance measurements obtained under different conditions. The following formula was used with a sampling rate of 40 kHz:


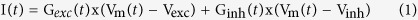


where I is the total current injected at time *t*; G_exc_ is the excitatory conductance at time *t*, G_inh_ is the inhibitory conductance at time *t*, V_m_ is the membrane potential at time *t*, V_exc_ is the reversal potential of excitatory currents and V_inh_ is the reversal potential of inhibitory currents.

The magnitudes of the excitatory and inhibitory synaptic conductances were scaled by a common factor but their ratio was kept constant in order to evoke responses consistent with those evoked by visual stimulation. A small constant current (<- 200 pA) was injected to keep resting potential between −65 and −70 mV if required.

### Tissue processing and morphology

At the end of each experiment cells were visualised with fluorescent light for initial morphological identification. Subsequently, tissue was fixed with paraformaldehyde (4% in 0.1 M phosphate buffer) and processed for immunocytochemical staining of Lucifer Yellow. Fixed retinae were incubated in primary anti-Lucifer Yellow rabbit IgG (1:10,000, Invitrogen) for five days, then in secondary goat anti-rabbit IgG (1:500, Invitrogen) overnight. Retinae were then wet mounted with Fluorescent Preserving Media, coverslipped, and sealed with nail polish. Ganglion cells were then visualised under a Leica Spec-II confocal microscope and morphologically reconstructed (see Figs 1a and 3a).

### Data analysis and statistics

Data analysis was carried out using custom written routines in IGOR (Wavemetrics, Lake Oswego, OR) and MATLAB (MathWorks, Natick, MA). Spikes were quantified by detecting the location of maxima calculated from the smooth first and second derivatives of the membrane potential signal and comparing it to a threshold. If the amplitude of that point was larger than threshold (usually over 0 mV), it was considered to be a spike. Spontaneous activity (0.9 ± 0.3 Hz) was observed in 14 out of the 33 RGCs recorded from and, when present, was subtracted from the stimulus-evoked responses. The amplitude of the membrane potential depolarisation (excitatory postsynaptic potential, EPSP) was calculated after removing spikes by linear interpolation of the membrane potential signal 4 ms before each spike and ~10 ms after each spike (as in Demb *et al.*^46^). EPSP amplitude was calculated as the difference between a baseline averaging 200 ms before the EPSP and the average membrane potential across 40 ms following peak responses. Rate of rise of the EPSP was calculated between 10 and 90% of the amplitude of the rising phase of the EPSPs. Inter-spike intervals were also measured.

A suppression index (SI) was calculated to estimate the degree of inhibition on the peak response, achieved by injecting currents composed of conductances measured with the largest spot:


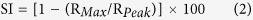


where R_*Max*_ is the response elicited with current injection based on conductances measured with the largest spot and R_*Peak*_ is the response elicited with current injection based on conductances that produce the peak response (optimal size). An index of 100 indicates that injection of currents based on conductances measured with large spots abolished response completely and an index of 0 indicates that they did not reduce response.

A non-parametric Mann-Whitney U-test was used to assess significant differences between two sets of data for individual cells. Wilcoxon matched-pairs signed-rank test (Wilcoxon test) was used for pair-comparisons of populations of cells in different conditions. A two-tailed criterion was used for these tests and significance was taken when P ≤ 0.05. Statistical analyses were made using MATLAB (MathWorks Inc., Natick, MA, USA). Values in the text represent mean ± SD (standard deviation), whereas data and error bars on plots represent mean ± SEM (standard error of the mean). Comparison of these responses allowed us to establish the relationship between the integrated input that the cell receives and its output response, providing critical information about the functional role of inhibition in spatial tuning and gain control.

## Additional Information

**How to cite this article**: Huang, J. Y. and Protti, D. A. The impact of inhibitory mechanisms in the inner retina on spatial tuning of RGCs. *Sci. Rep.* **6**, 21966; doi: 10.1038/srep21966 (2016).

## Figures and Tables

**Table 1:** Response properties of mouse RGC types.

Cell type	EPSP amplitude (mV)	Frequency (spikes/s)	Rise Time (mV/ms)
A (n = 14)	18.1 ± 1.6	126.3 ± 14	0.77 ± 0.07
B (n = 6)	21.6 ± 2.4	137.1 ± 14.6	0.92 ± 0.08
C (n = 5)	24.9 ± 2.9	164.9 ± 37	1.09 ± 0.1
Unclassified (n = 8)	18.8 ± 1.6	130 ± 12.5	0.89 ± 0.05

Quantification of responses from injections to currents generated with conductances that produced peak responses (optimal size). Values are means ± SEM.
